# Annotations

**Published:** 1891-08-22

**Authors:** 


### Annotations.

**Out of the World.**

This is holiday time for those who have opportunity and wisdom therewith to make holiday. Unfortunately, there are many people not generally counted insane, whose idea of a summer holiday is to live elsewhere the life they usually lead at home. They call it a little change—and rightly too, for the change is, indeed, but small. But what most people want who work hard during ten or eleven months of the year is a change, thorough and complete, not merely of air and scene, but of habits, associations—even thoughts. We are all more or less bound by conventions; we cannot for the sake of our daily bread too deeply outrage the prejudices of the Browns, Jones, and Robinsons who supply it. But all the more joy to escape to some lonely isle or distant moorland where the natural man may cast off his tall hat, and the natural woman cut her skirts to a length and width that permit of climbing hills and scrambling over stiles, where all humanity may abjure gloves from the day of arrival to the last moment of departure. "But it is so lonely," some would say; "there is such a lack of occupation and amusement." Therein they err, mistaking the thing called society for true companionship, and the daily round for all possibilities of interest. There are wiser men in out-of-the-way hamlets than the dwellers in towns are apt to think. Britain could hardly be the great country it is if all its great men were the few score who write for the magazines and speak in the House of Commons. Wisdom is justified of all her children, of farmers and fishermen, weather-wise and learned in the ways of beast and bird, as well as of smart journalists and voluble M.P.'s. Then for occupation—is it nothing to go out searching for mushrooms on dewy hilltops in the morning, or fishing as the crimson sunset turns to grey at night, feeling all the more interested in the self-imposed task if one can bring oneself to think that a meal depends on its success. We cannot all be dukes and millionaires and have deer forests at our command; but most of us can find some nook where life may flow in something like its primal channel, and where we may for a little while realise that ideal of man—strong, fearless, made in the image of God—whose memory lingers in every country in some old tradition as the patriarch and pattern of all.

**Nurses in Workhouses.**

There can be no doubt that the Lords' Committee on Hospitals would be well advised if they were to take up the subject of the treatment of the sick poor in workhouses as apart from workhouse infirmaries. We are assured that gross cruelties are still perpetrated in the sick wards of many workhouses to-day which are of so serious a character, that could the public attention be fixed upon them an outcry would arise throughout the whole country which would compel the retirement of many guardians, and lead to drastic reforms. Such reforms are called for not only on the ground of efficiency, but on the stronger ground of humanity itself. The average guardian's one idea is that anything is good enough for workhouses, and his sole object seems to be to cut down expenditure, though some of them, it is to be feared, are very apt in giving out contracts to favour their friends, and so to deprive the paupers of the best of rations of the common kind usually supplied. In the matter of nursing, the sick wards of the workhouses are, as a whole, lamentably deficient. It is not too strong a statement to say that where there are pauper nurses there cruelty and neglect mainly predominate in the treatment of the sick. Why the Local Government Board should not endow its Inspectors with sufficient power and courage to root out the crying evils we refer to passes general comprehension. The Strand Union is notoriously bad, and has an evil reputation, which is proved to be deserved by recent inquest revelations. No doubt the methods of the Local Government Board require reform, or why did they appoint the Inspector of the district to preside over the recent Committee of Enquiry into the management of the Eastern Hospital, seeing that the Chairman of that enquiry was practically placed upon trial by the proceedings, as they related to the condition of the internal management of this institution for the efficiency of which the said Inspector was himself responsible? At a recent conference at Gilsland several of the medical officers of workhouses situated in the four northern counties spoke of the great hardship of pauper nursing to the poor, and it was the unanimous opinion that the public interest demanded the cutting out, root and branch, of pauper nursing once and for all. How great is the evil may be judged from the fact that the pauper nurse is never efficient, and that, as Dr. Ainsley proved by statistics, in twenty-two workhouse infirmaries the nursing staff consists of one nurse to forty patients. How gross is the mismanagement which allows such a state of things will be realised when we state that the managers of the London hospitals have been charged with neglect before the Lords' Committee, because they did not provide more than one nurse for each ten patients in their wards! A moment's reflection will show any thoughtful person that to place one nurse in charge of forty patients is to prove that the authorities responsible for such a system are unworthy of public confidence and ought to be promptly removed from office. There is small merit in providing efficiently for those who can help themselves. In the case of the pauper poor, who are ill and disabled, the responsibility upon the individual guardians who permit the disgraceful state of affairs to exist to which we here call attention, is too great to need further comment. If the ratepayers, knowing the facts, neglect to enforce reforms, they must share the responsibility with the guardians, who prove by the maintenance of such maladministration that they are incapable, incompetent, and altogether inhuman. We could wish that it were possible to assign a certain class of guardians, when ill, to a pauper bed in the sick ward of their own workhouse, and to place them in the charge of the pauper nurse they provide, and make responsible for the nursing of forty patients.

**The Cause of Tetanus.**

Tetanus, or, as most people call it, lockjaw, remains one of the most mysterious diseases known to science, so slight a cause may produce it, and so cruel are its effects. It seems incredible that a slight cut or unimportant bruise should develop a deadly ailment, and many people were inclined to think that it was due to some purely constitutional predisposition, to "something in the blood," or other causes as mystic and alarming. To a certain extent popular opinion was right, for Dr. Sims Woodhead's book on "Bacteria and their Products" shows that one of the first conditions of the development of the tetanus bacillus is the absence of oxygen. When, therefore, the system is starved of that first essential of health, there is something in the blood—to speak more accurately, a lack of something—which gives the tetanus poison a better chance than it would otherwise have. But strong men contract lockjaw—grooms, gardeners, soldiers, all whose occupations are of a sufficiently healthy nature. The truth seems to be that there is a specific tetanus poison in the soil, more abundant in some places than others, but rarely wholly absent. It is contact with this impregnated soil that converts a slight abrasion into a deadly wound. A barefooted child may be poisoned after a slight cut; a groom may die after a kick from a horse's shoe, not because the bruise thus inflicted is severe, but because the horse's shoes are dirty; a gardener may carry about on his fingers an enemy that wants only the slightest loophole to enter his system. Soldiers are more liable to tetanus when camping out, and in hot countries where the inhabitants sleep out of doors it is much more common than in our climate. Savages, more scientific than our scientists, know the deadly power of the soil when they dip their poisoned arrows, destined to cause convulsions in those wounded by them, in earth taken from a mangrove swamp. Fortunate it is that oxygen is the foe of the tetanus poison. Even the poisoned arrows lose their virulence after long exposure to the air, and in healthy organisms, where the great vessels are continually distributing ample supplies of oxygenated blood, the sound tissue refuses to yield to the disease. Pure air and pure water are the enemies of tetanus, as of most other ailments, and fight a good fight against the lurking dangers of the soil.

